# Supplementary figures and images for: ForestForward: visualizing and accessing integrated world forest data from the last 50 years
Source: Database (Oxford). 2025 Mar 3;2025:baaf018. doi: 10.1093/database/baaf018 (PMC11879282; doi:10.1093/database/baaf018)

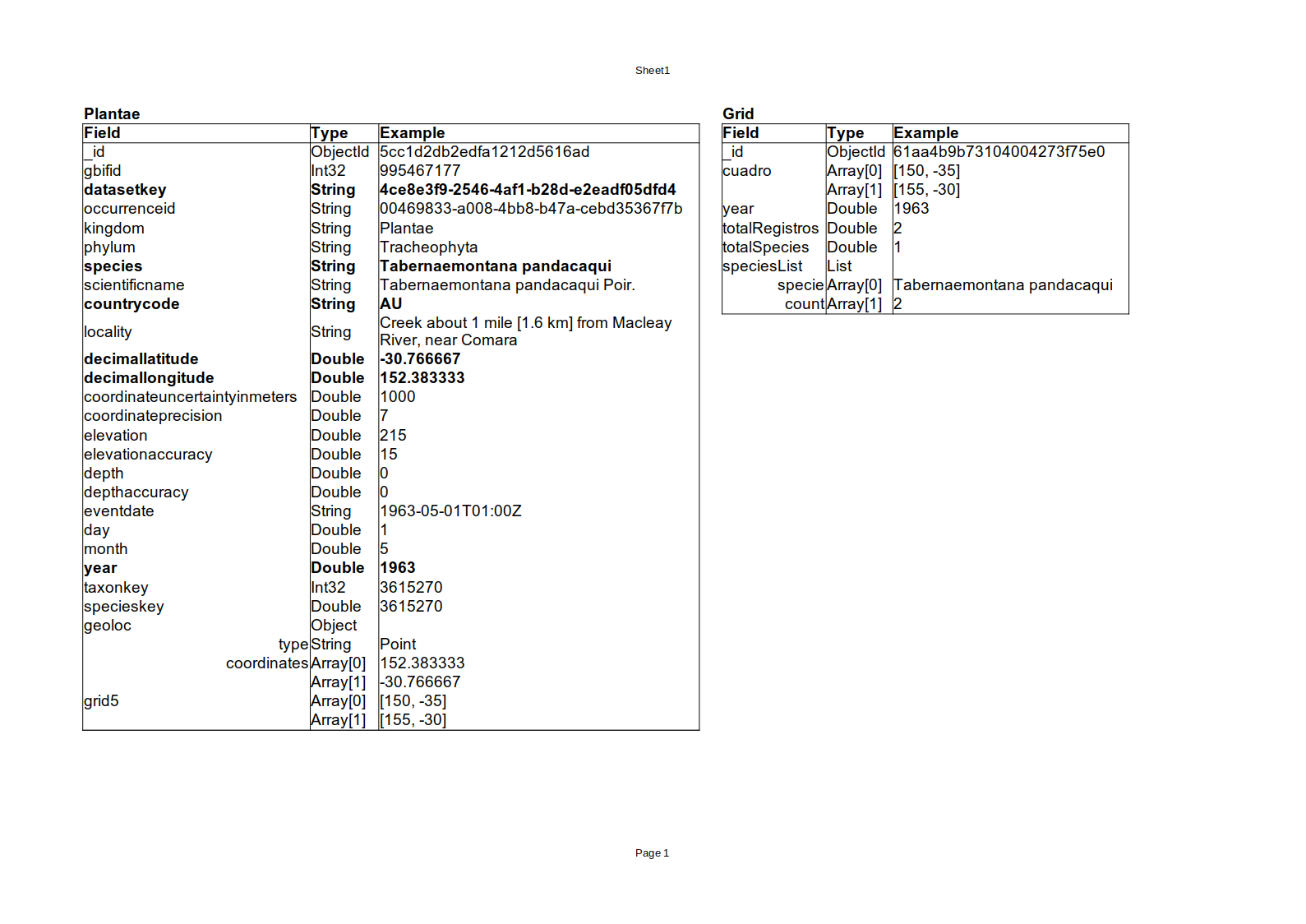

Supplement: baaf018_Supp [file baaf018_supp.zip › suppl_data/Suplementary1.png]
